# Brain Energy Metabolism: Astrocytes in Neurodegenerative Diseases

**DOI:** 10.1111/cns.13982

**Published:** 2022-10-03

**Authors:** Zhenlei Chen, Ziqi Yuan, Shangchen Yang, Yufei Zhu, Maoqiang Xue, Jie Zhang, Lige Leng

**Affiliations:** ^1^ Fujian Provincial Key Laboratory of Neurodegenerative Disease and Aging Research Institute of Neuroscience, School of Medicine, Xiamen University Xiamen China; ^2^ Department of Basic Medical Science, School of Medicine Xiamen University Xiamen China

**Keywords:** Alzheimer's diseases, astrocytes, metabolism, microglia, neuron, Parkinson disease

## Abstract

Astrocytes are the most abundant cells in the brain. They have many important functions in the central nervous system (CNS), including the maintenance of glutamate and ion homeostasis, the elimination of oxidative stress, energy storage in glycogen, tissue repair, regulating synaptic activity by releasing neurotransmitters, and participating in synaptic formation. Astrocytes have special highly ramified structure. Their branches contact with synapses of neurons inwardly, with fine structure and wrapping synapses; their feet contact with blood vessels of brain parenchyma outward, almost wrapping the whole brain. The adjacent astrocytes rarely overlap and communicate with each other through gap junction channels. The ideal location of astrocytes enables them to sense the weak changes of their surroundings and provide the structural basis for the energy supply of neurons. Neurons and astrocytes are closely coupled units of energy metabolism in the brain. Neurons consume a lot of ATPs in the process of neurotransmission. Astrocytes provide metabolic substrates for neurons, maintain high activity of neuron, and facilitate information transmission of neurons. This article reviews the characteristics of glucose metabolism, lipid metabolism, and amino acid metabolism of astrocytes. The metabolic interactions between astrocytes and neurons, astrocytes and microglia were also detailed discussed. Finally, we classified analyzed the role of metabolic disorder of astrocytes in the occurrence and development of neurodegenerative diseases.

## Graphical abstract

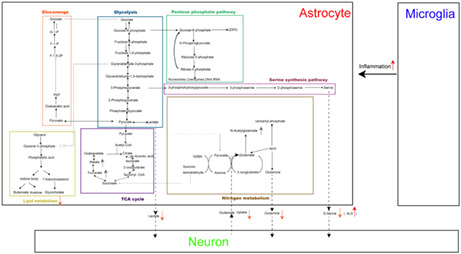

## 1 INTRODUCTION

Astrocytes are the greatest number and special ramified cell‐type in CNS. They have several functions: their processes and cerebral vessels form the blood–brain barrier (BBB); their synapses represent the third element of the tripartite synapse; they provide a certain degree of metabolic supplement for neurons.

Astrocytes provide essential metabolic support for neighboring neurons and other cell types, and protect their neighboring cells by ingesting excessive glutamate and K^+^ and releasing growth factors, lactic acid, glutamine, mitogen, and other necessary chemical messengers. With the development of aging, injury, and disease, astrocytes will undergo significant morphological and molecular phenotypic changes, among which the most extensive features are cell hypertrophy and the upregulation of intermediate filament protein GFAP. Morphological hypertrophy of astrocytes is one of the main pathologies identified by Alois Alzheimer in 1910.^1^ It is now considered as a marker of AD and most other forms of brain injury and chronic neurodegeneration. Although the reactive astrocytes of AD have a long history and significant performance, they are not as deeply studied as other major cell types in the brain, neuron, and microglia. Therefore, the effect of reactive astrocytes on the pathophysiology of AD is still unclear and speculated. In recent years, the research on energy metabolism of brain has changed from single center of neuron to three‐way metabolic coupling of neuron, astrocyte, and microglia. More attention has been paid to the energy metabolism cooperation among neurons, astrocytes, and microglia. Large number of studies have found that the mRNA expression of various metabolic enzymes in astrocytes changes in neurodegenerative diseases.^2^, ^3^, ^4^, ^5^ The material transfer and interaction among the three are of great significance to maintain the energy metabolism in the brain under various physiological and pathological conditions.

In this review, we discussed the characteristics of glucose metabolism, lipid metabolism, and amino acid metabolism of astrocytes in detail. The metabolic interactions between astrocytes and neurons, astrocytes, and microglia were also discussed. Finally, we analyzed the role of metabolic disorders of astrocytes in the occurrence and development of neurodegenerative diseases.

## 2 INTRODUCTION OF ASTROCYTES

Glial cells in the central nervous system (CNS) mainly contain microglia, oligodendrocytes, and the most diverse astrocytes. The concept of astrocyte was first put forward by Rudolf Virchow in the 19th century.^6^ Later, Camillo Golgi used silver chromate staining to visualize the morphology of astrocytes.^7^ Astrocytes were quickly divided into two basic morphological subtypes: protoplast type and fibrous type.^8^ Protoplast astrocytes are usually found in gray matter, while fibrous astrocytes are commonly found in white matter of CNS. Due to the deepening of neuroanatomy and morphological analysis, astrocytes are divided into four types: interlayer type, varicose type, protoplast type, and fibrous type.^9^

With the progress of transcriptome analysis tools, two types of reactive astrocytes were described in 2012. Compelling evidence suggests that two injury mouse models, neuroinflammation induced by lipopolysaccharide (LPS) and ischemic stroke induced by MCAO, identified two stimulus specific reactive astrocyte subtypes with unique transcriptome characteristics, called A1 astrocytes (detrimental) and A2 astrocytes (beneficial), respectively.^10^ A1 astrocytes can secrete a harmful neurotoxin, which can induce apoptosis of neurons and oligodendrocytes.^11^ However, transcriptomic analysis of A2 showed upregulated the expression of anti‐inflammatory genes and increased phagocytosis of microglia to protect neurons from Aβ toxicity.^12^ The latest development of single cell transcriptomics has led to the identification of five astrocyte subsets (AST) in the mouse nervous system, showing various forms and functions. AST1 is defined as the high expression of *Gfap* and *Agt*, and AST2 is defined as the deletion of *Agt* and the high expression of *Unc13c* and *Slc1a3*. The distribution of AST3 is predicted by theexpression of *Agt* and low expression of *Unc13c* and *Gfap*. While AST4 is identified by high expression levels of *Frzb*, *Ascl1*, and *Slc1a3*. Finally, AST5 is identified by low expression of *Ogt* and high expression of *Fam107a* and *Slc1a3*. AST1 and AST4 are mainly distributed in hippocampus, AST2 is mainly distributed in cortex, AST3 and AST5 are evenly distributed between brain regions.^13^ Among them, AST2 and AST3 differentially expressed genes related to neurotransmission in the cerebral cortex. AST2 is rich in transcripts related to glutamate neurotransmission.^14^ AST3, in contrast, is rich in transcripts associated with GABAergic neurotransmission.^15^, ^16^ In addition, AST2 is rich in chordin‐like 1 (Chrd1), which is an important factor in stabilizing synapses.^17^

## 3 METABOLISM OF ASTROCYTES

### 3.1 Carbohydrate metabolism of astrocytes

While the brain consumes a lot of energy, it is highly dependent upon the uninterrupted supply of energy substrates from the circulation. Glucose is the main energy source of the brain, which can be directly metabolized or stored in the form of glycogen. The loss of glucose of brain can lead to neurological disorders, unconsciousness, even coma within minutes.^18^ However, under specific conditions, brain cells can effectively utilize various energy substrates besides glucose, including lactic acid, pyruvate, glutamate, and glutamine.^19^ Most of these metabolites are formed endogenously by using glucose as carbon source. As brain metabolism is a process of compartmentalization, these metabolites undergo complex intercellular transport in the brain.

Through glucose transporter (GLUT) subtypes with different kinetic properties, glucose shuttles back and forth in brain cells reversibly from arterial blood through endothelial cell membrane.^20^ GLUT1 is mainly located in endothelial cells and astrocytes, while GLUT3 and GLUT4 are mainly located in neurons. After glucose enters the cell, it is phosphorylated by hexokinase (HK) to produce glucose‐6‐phosphate. Like other organs, glucose‐6‐phosphate can be processed through different metabolic pathways, mainly in three ways^21^: the first is glycolysis, which leads to lactic acid production or mitochondrial metabolism, and the second is pentose phosphate pathway (PPP). The third is oxidative phosphorylation (OXPHOS). The fourth, there is glycogenesis, which is only in astrocytes.

Firstly, glucose‐6‐phosphate can be metabolized by glycolysis to produce two pyruvate molecules, ATP and NADH. Pyruvate can enter mitochondria and produce ATP and CO_2_ while consuming oxygen through the tricarboxylic acid cycle (TCA) and oxidative phosphorylation metabolism. Pyruvate can be reduced to lactic acid by lactate dehydrogenase (LDH). Lactic acid can be released in vitro through monocarboxylate transporter (MCT). Compared with glycolysis (2 ATP), Pyruvate produced by glycolysis can enter the tricarboxylic acid cycle (TCA) and be completely oxidized within the mitochondria to produce more energy in the form of ATP (30–34 ATP). Alternatively, glucose‐6‐phosphate can be treated by PPP to produce NADPH equivalent. It is noteworthy that PPP and glycolysis are associated at glyceraldehyde‐3‐phosphate and fructose‐6‐phosphate levels. Finally, in astrocytes, glucose‐6‐phosphate can also be used as a glycogen storage unit.^22^

Importantly, both astrocytes and neurons have the ability to completely oxidize glucose and/or lactate, which is consistent with the observation that both cell types have the same amounts of mitochondria.^23^ Although astrocytes show lower oxidative metabolic rates than neurons, they greedily absorb glucose and typically exhibit high glycolysis rates.^24^ Most glucose that entering the astrocyte glycolysis pathway is released into lactate in extracellular space.^25^ Lactate can be exported from astrocytes through monocarboxylate transporter 1 or 4 (MCT1/4) and transported to neurons through MCT2.^20^ In neurons, astrocyte‐derived lactic acid is converted back to pyruvate and transported to mitochondria to produce ATP through the TCA cycle.^21^ LDH, of which isoenzyme LDH‐5 is found to be specific to astrocytes, is an important metabolic enzyme in the process of lactate shuttle.^22^ The lactate shuttle from astrocytes to neurons plays an important role in the energy supply of neurons. This is the well‐known astrocyte lactate shuttle hypothesis published by Pellerin and Magistretti in 1994, in which lactic acid is transferred from astrocytes to neurons in conjunction with the neurotransmitter glutamine.^23^ According to this metabolic mechanism, astrocytes are in the perfect position of balanced metabolism. The glycolytic properties of astrocytes and their preference for the production of lactic acid rather than the entry of pyruvate in the TCA cycle are the result of a specific gene expression profile, which involve a variety of enzyme and transporter phenotypes in the brain.^26^ Studies have shown that lactic acid produced by glycolysis of astrocytes is essential for the potential regulation of long‐term memory formation and the molecular changes required for long‐term memory formation.^27^ Therefore, glucose metabolized by glycolysis in astrocyte can promote and meet high energy requirements related to cellular changes in learning, memory formation, and memory storage. But neurons are not entirely dependent on the lactic acid supplied by astrocytes. Lundgaard et al. and Diaz‐Garcia et al.^28^, ^29^ found that glucose is metabolized in situ by neurons in an activity‐dependent manner in awake mice. To be precise, neurons do metabolize lactic acid, but lactic acid is more like an “opportunistic” substrate that, if present, will indeed help support energy metabolism.^30^

Other studies have suggested that^31^ the high energy requirements of stimulated neurons are supported by astrocytes, which provide neurons with lactic acid produced by aerobic glycolysis, thus providing the energy required for the activity to induce neuron function. A lot of evidences agree that neurons have high oxidative activity, indicating that glycolysis mainly occurs in astrocytes.^23^, ^32^ That is to say that astrocytes are mainly glycolytic cells, not neurons.

Although the level of glycogen in the CNS is relatively low compared with the surrounding tissues, it is the largest energy reserve of the brain. Interestingly, at the cellular level, glycogen has been found to be almost confined to astrocytes in the adult brain.^33^ It represents an advantageous form of glucose storage because it can be metabolized rapidly without the need for ATP, and unlike fatty acids, it can produce ATP under anaerobic conditions.

In addition to its role as an emergency energy reserve, glycogen in brain has many important physiological functions. Glycogen content is dynamically controlled by neurotransmitters, neurohumoral factors, and local energy state.^34^ It was also found that decreased neuronal activity observed during sleep and anesthesia was associated with increased brain glycogen levels, consistent with the concept of awake brain utilization of glycogen.^35^ In addition, the increase of neuronal activity induced by sensory stimulation is related to the decrease of glycogen level in the activated area, indicating that there is a close coupling between neuronal activity and glycogen mobilization.^36^ Furthermore, astrocyte glycogen mobilization is possibly related to the transfer of lactic acid from astrocytes to neurons, as the absolutely necessary part of maintaining neuronal activity^37^ and glutamatergic synaptic transmission.^38^ These strong evidences indicate that glycogen plays a key and indispensable role in normal brain function and activity.

### 3.2 Lipid metabolism of astrocytes

Lipids are important components of nerve cell membranes, which participates in brain function in different ways. It is indispensable for astrocytes to participate in functions, such as energy generation, membrane fluidity, and intercellular signal conduction. It has been found that the lipid droplets stored by astrocytes play an essential physiological and protective role in the CNS.^39^

Interestingly, the brain is considered autonomous in lipid metabolism as the BBB prevents lipid from entering the brain.^40^ Neurons have a limited capacity for lipid metabolism, so they must take enough lipids from the external environment.^41^ Astrocytes have strong lipid metabolism enzyme activity, and manage the nutritional support needed by brain for neuron consumption.^42^ Therefore, it is generally believed that the lipids produced by astrocytes are absorbed by neurons to support the formation and function of synapses. In recent years, the synthesis of cholesterol and fatty acids in astrocyte has been studied, which depends on the sterol regulatory element binding protein (SREBPs).^43^ SREBPs regulate the transcription activation of fatty acid and cholesterol metabolism‐related genes,^44^ and the transcription factors mainly depend on the post‐translation activation of SREBP cleavage activator (SCAP) involved in sterol sensor. It is proved that astrocytes are the main SREBP expression cell types in hippocampus, and the decrease of SREBP activity in astrocytes leads to the damage of pre synaptic terminal function and synaptic plasticity.^45^ In summary, these studies show that lipids produced by astrocyte is significant in synaptic development and brain function.

Cholesterol and unsaturated fatty acids are enriched in synaptic membrane, which specially affect a series of biochemical processes, including membrane fluidity, vesicle formation and fusion, ion channel function, and contribute to the formation of special micro areas of cell communication.^46^ Cholesterol is the main form of cholesterol lipid in the brain. Among all the lipids in astrocytes, cholesterol may play the first part in astrocyte structure. Cholesterol helps regulate the elasticity of cell membrane by interacting with nearby phospholipids,^47^ and also includes lipid raft formation, glucose transport, and inflammatory signals.^48^ It is necessary to form synaptic vesicles before synapses and to form neurotransmitter receptors after synapses. Because of the existence of BBB, astrocytes' cholesterol metabolism is independent of other cholesterol metabolism in vivo. In addition, astrocytes had higher cholesterol level and lower expression of cholesterol synthetase in neurons. Therefore, astrocytes were considered to be the main place for cholesterol synthesis, mainly occurred in ATP‐dependent process in endoplasmic reticulum.^49^ Cholesterol is produced through a complex process, which can be roughly divided into two biochemical pathways: Bloch pathway and the Kandutsch–Russell pathway. And the research has found that astrocytes synthesized cholesterol mainly through Bloch pathway, which is dominant for cholesterol biosynthesis in mouse models.^50^

Astrocytes respond to ischemia by AMP‐activated protein kinase (AMPK) in vitro to promote the production of ketone body. The ketone body produced by astrocytes are derived from fatty acids, which may be used as the energy substrate of TCA cycle, rather than l‐lactate, because pyruvate dehydrogenase is susceptible to ischemia.^51^ Therefore, fatty acids can be used to initially produce ketone body from astrocytes and then exchange between astrocytes and neurons through monocarboxylate transporter to generate energy through TCA cycle.

Sphingomyelin is a kind of lipid characterized by sphingosine skeleton. This includes a range of lipids, including ceramide, sphingosine, and sphingomyelin. The lipids produced by astrocytes are supplied to neurons and oligodendrocytes as part of synaptic and myelin membranes.^38^ Compared with the oxidative metabolism of glucose in resting brain, lipid oxidation metabolism was inhibited,^52^ but it was also regulated by the interaction between astrocytes and neurons. Sphingolipids have some functions in astrocytes, including inflammatory regulation.^53^ At present, it has been found that normal production of ganglioside by astrocytes can also promote the growth of neurites, regulate the inflammation of neurons, and stabilize the interaction between neurons and glial cells.^54^

Apolipoprotein E (ApoE) is a lipid carrier in the CNS. ApoE‐positive vesicles are swallowed by adjacent astrocytes, and are degraded into free fatty acids (FAS) by lysosome.^55^ Then the free FAS adhere to lipid drops (LDS), which will mediate metabolism through mitochondria β‐Oxidation and phosphorylation. Astrocytes absorb these ApoE positive LDS through endocytosis, and provide energy for mitochondrial‐mediated apoptosis β‐Oxidation, as well as this coupling of lipid metabolism between neurons and astrocytes, protects neurons from fatty acid toxicity.^56^ Astrocytes are not only the key cells to ingest and export FAS and apolipoprotein, but also the main cell group that mediates apoptosis β‐Oxidation of FAS in the brain.^57^ Astrocytes transport lipids from BBB to neurons by binding and internalizing the permeable FAS of BBB from endothelial cells, and by the action of ATP binding box transporters (such as ABCA‐1), and loading ApoE into the cargo.^58^ Brain is one of the largest sites for ApoE synthesis, second only to liver.^59^ Most of the ApoE is derived from astrocytes, which are bound to receptors in the low‐density lipoprotein (LDL) receptor family, which are expressed in astrocytes and neurons.^60^ Therefore, astrocytes are the vital parts of the lipid uptake‐ApoE‐lipidation axis, and are essential to maintain the lipid homeostasis and normal neuronal function in the brain. The role of ApoE from astrocyte and microglia in tau lesions is unclear. A recent study found that astrocyte derived ApoE particles were much larger than microglia, and thus contained more lipids. It suggesting that the two cell‐derived ApoE have different roles and functions.^61^ Holtzman DM et al.^60^ found that astrocytic ApoE4 removal decreasing disease‐associated gene signatures in neurons, oligodendrocytes, astrocytes, and microglia. Removal of astrocytic ApoE4 decreased tau‐induced synaptic loss and microglial phagocytosis of synaptic elements, suggesting a key role for astrocytic ApoE in synaptic degeneration.^62^

### 3.3 Amino acid metabolism of astrocytes

Amino acids have various functions in astrocytes. In addition to their roles in protein biosynthesis, they also represent components of several other biosynthetic pathways. Astrocytes are obviously involved in the metabolism of all amino acids, but the main research direction is glutamate and GABA, two main neuroactive amino acids, which play a role in astrocytes.^63^ Therefore, we focus on the glutamate/GABA‐glutamine cycle in astrocytes. Glutamate/GABA‐glutamine cycle is a process in which the two neurotransmitters are removed from synaptic cleft by astrocyte absorption, then transformed into glutamine. The newly‐synthesized glutamine is transferred into neurons to re‐synthesize neurotransmitters.^64^, ^65^ Glutamine is abundant in the CNS. It is the precursor of many neurotransmitter amino acids, including the excitatory amino acids, glutamic and aspartic acid, and the inhibitory amino acids, γ‐amino butyric acid (GABA).^66^

Studies have shown that different responses in this cycle are clearly divided between astrocytes and neurons.^63^ For example, Glutamine synthetase (Gs) is an enzyme that transforms glutamate into glutamine, which is expressed only in astrocytes.^65^ Glutamine is converted into glutamate by phosphate activated glutaminase (PAG), and its expression in neurons is much higher than that in astrocytes.^67^ In addition, GABA is synthesized by glutamic acid decarboxylases (GADs), which are widely present in GABAergic neurons.^68^

In conclusion, glutamates are released into synaptic cleft in glutamate neurons, absorbed by astrocytes, and then transformed into glutamines by GS. The newly‐synthesized glutamines are transported to glutamate neurons, and then transformed into glutamates by PAG to participate in the next cycle. In GABAergic neurons, GABA is removed from synaptic cleft by astrocytes. In astrocytes, GABA and α‐ketoglutarate are converted to ammonia, then produced succinic acid. Glutamine is synthesized from succinic acid through tricarboxylic acid cycle (TCA), including citric acid formed by oxaloacetic acid and acetyl‐CoA and synthesized of ketoglutarate and glutamate. The newly‐synthesized Glutamine is transferred to GABAergic neurons and hydrolyzed to produce Glu, some of them can be decarboxylate to GABA.^69^

Although the glutamate/GABA glutamine cycle is widely accepted, it is the result of deliberate simplification. For example, the cycle does not take exogenous glutamate into account. The studies have proved that glutamate not only comes from neurotransmitters, but also from glucose, lactic acid and so on.^70^ Even some branched‐chain amino acids can be used as the source of glutamic acid.^63^ Moreover, astrocytes can not only synthesize glutamine, but also oxidize glutamine.^71^

## 4 METABOLIC INTERACTION OF ASTROCYTES

### 4.1 Metabolic interaction between astrocytes and neurons

Lactate shuttle between astrocytes and neurons is essential for maintaining normal brain function. Lactate not only provides energy to neurons, but also mediates long‐term memory and increases neuronal excitability.^72^

Glutamate is an excitatory neurotransmitter in the brain, and its excessive aggregation in the brain can easily stimulate glutamatergic receptors (GluRs) on neurons and cause neurotoxic damage.^21^ Glutamatergic neurons excitedly release glutamate into the extracellular space and absorb it into the cells through astrocyte specific Na^+^‐dependent GLT‐1(glutamate transporter 1) and GLAST (glutamate/aspartate transporter), with three Na^+^ ions entering the cells. Because the above reaction can disturb the ion balance inside and outside the cell, it can activate Na^+^/K^+^ ATP pump and increase ATP consumption.^73^ Glutamic acid in astrocytes is converted to glutamine under GS catalysis, which is also a process of energy consumption. The glutamine produced shuttles back and forth into neurons and produces glutamate under the action of glutaminase (GLSs). Glutamate shuttling triggers energy consumption, which can increase the glucose absorption of astrocytes, and then lead to the increase of glycolysis and glycogen decomposition, the increase of lactic acid production, shuttling into neurons, as an energy substrate to provide ATP for neuron activation.^73^ Astrocytes are also efficient at consuming glutamate to offset the energy expend in glutamine synthesis.^74^ The pyruvate carboxylase of astrocytes converts glucose into glutamate to supplement the amount of glutamate in the brain.^75^ The latter can also be converted into α‐ketoglutarate to enter the TCA cycle for oxidative metabolism.

Astrocytes convert glucose to l‐serine through a series of enzymatic reactions such as 3‐phosphoglycerate dehydrogenase (3PGDH). Selective knockout of astrocytes 3PGDH can reduce the levels of l‐serine and d‐serine in neurons.^76^ n‐methyl‐d‐aspartate (NMDA) is a glutamate receptor. The agonist is d‐serine, which binds with glutamate to open its channel and activate glutamatergic neurons. In astrocytes, l‐serine reaches the extracellular matrix through ASCT1 (*Slc1a4*) transporter, enters neurons through Asc‐1 (*Slc7a10*) transporter, and transforms into d‐serine, or serine shuttle. Astrocyte ASCT1 was knocked out, l‐serine transport was impaired, and l‐serine and d‐serine were expressed at low levels in neurons.^77^ However, glycine in astrocytes is not sensitive to ASCT1. When ASCT1 is injured, a large number of astrocytes can penetrate into neurons and generate l‐serine under the catalysis of serine hydroxymethyl transferase.^77^ ASCT1 knockout mice cause serine deficiency and extensive amino acid metabolic disorders (alanine, threonine and glycine), which lead to exercise impairment and learning and emotional behavior defects in mice.^77^ Astrocytes can also use d‐serine synthase serine racemase (SRR) to transfer l‐serine.

However, the synthesis of d‐serine is less than that of neurons. This is mainly due to the fact that glyceraldehyde 3‐phosphate dehydrogenase (GAPDH) produced by astrocyte glycolysis can bind to SRR and inhibit its activation, and the product NADH can also allosterically destroy the stability of SRR. ATP produced by glycolysis can also amplify the interaction between GAPDH and SRR, and finally make SRR stable. In the inactive state, the production of d‐serine decreased.^78^

When neurons were activated, they released norepinephrine (NA), vasoactive intestinal peptide (VIP), and adenylate cyclase receptor on the surface of astrocytes. cAMP concentration increased and astrocytes glycogen metabolism and glycolysis were stimulated. cAMP‐dependent protein kinase (AMPK) phosphorylates acetyl coenzyme A carboxylase (ACC), which reduces the activity of ACC, resulting in the reduction of malonyl coenzyme a synthesis and the inhibition of carnitine palmitoyl transferase (CPT‐I). CPT‐I carries fatty acids into mitochondria for β oxidation and produces ketone bodies, which are used as substrates for neuronal oxidation and biosynthesis.^79^ During ischemia/hypoxia, the ratio of AMP/ATP in astrocytes increased, AMPK activity increased, and ACC activity was inhibited. As mentioned above, ketone body production increased, and neurons preferred to use ketone body as energy substrate.^79^ Hypoxia can also increase the non‐oxidative metabolic pathway of non‐esterified fatty acids in astrocytes, increase the production of ceramide, activate extracellular signal regulated kinase (ERK), and lead to neuronal apoptosis, while AMPK activation can increase the β oxidation of fatty acids and the production of ketone bodies, reduce the de novo synthesis of ceramide, which is conducive to the survival of neurons.^79^ Therefore, ketogenesis can be used as the main metabolic regulation mechanism of brain protection.

Cholesterol is abundant in the brain and is an important part of cell membrane, synapse and myelin sheath. It is synthesized in astrocytes and regulated by 3‐hydroxy‐3‐methyl‐glutaryl coenzyme A reductase (HMGCR) and sterol regulatory element binding protein (SREBP2).^41^, ^80^ When brain cholesterol content is high, SREBP cleavage activating protein (SCAP) isolates SREBP2 precursor in endoplasmic reticulum; when cholesterol is needed, it can induce SREBP2 to shuttle to Golgi body, shear into transcriptional active form, and then translocate into nucleus, bind to sterol regulatory element DNA, activate cholesterol synthase transcription, and synthesize cholesterol.^80^ Cholesterol binds to LXR‐RXR, regulates the expression of efflux genes (*ApoE*, *ABCA1*, and *ABCG1*), transports lipoprotein granules and internalizes them into neurons. Cholesterol 24 hydroxylase (CYP46A1) is mainly expressed in neurons, catalyzing the degradation of cholesterol to 24 s hydroxycholesterol (24OHC), which reaches the blood through the BBB and enters the liver for further degradation.^41^ Defective cholesterol synthesis can lead to impaired neurite growth and changes in memory and motor behavior.^80^ Therefore, cholesterol synthesis and metabolism are very important for brain physiological function.

Maintenance and restoration of ion gradients dissipated by signaling processes, as well as uptake and recycling of neurotransmitters after neuronal firing, are the main brain energy needs.^81^ Unlike other mesenchymal cells from other organs, astrocytes are involved in the formation of the BBB, so the process of blood supplying nutrients to neurons requires the participation of astrocytes. And astrocytes are responsible for sensing changes in the energy demand of neurons, and are the essential contributors to vasomotor responses, participating in both vasoconstriction and vasodilation.^82^ Neurons do not metabolize lipid and could hardly perform gluconeogenesis, making them highly dependent on other supportive cells and external energy material supply, and highly susceptible for injury arising from energy supply failure, which results in two characteristics of astrocytes. One is that astrocytes are highly glycolytic.^21^ The other is its ability to regulate vasodilation and blood supply by sensing the energy demand of surrounding neurons. Increased astrocytic activity—reflected by increased astrocytic Ca^2+^ concentrations—led to the activation of glycolysis and an elevation of extracellular lactate and adenosine concentrations, particularly during lower‐oxygen conditions. High external lactate hinders PGE2 clearance, thus increasing extracellular PGE2, which induces arteriolar dilatation. In addition, adenosine released under low oxygen inhibits astrocyte‐mediated vasoconstrictions at the level of smooth muscle cells by blocking the effect of arachidonic acid.^83^ Recent study^84^ showed a line of the transgenic mice which express a step function type of light‐gated cation channel (channelrhodopsine‐2; ChR2) in astrocytes (Mlc1‐positive). Photo‐activation of ChR2‐expressing astrocytes resulted in a widespread increase in cerebral blood flow (CBF), extending to the non‐stimulated periphery. Such optogenetic manipulation may make it possible to regulate CBF supply by regulating astrocytes.

### 4.2 Metabolic interaction between astrocytes and microglia

Microglia are resident myeloid cells of the mammalian CNS, and colonized in CNS during early embryonic development. They are highly motile cells that interact with all cells of the CNS to regulate normal development, homeostasis, and general brain physiology. In the process of neurodegenerative diseases, the polarization and function of microglia are closely related to the disease process.

Molecular dialogue between microglia and astrocytes begins shortly after they begin to fill the brain parenchyma. Astrocytes and microglia actively regulate neuronal activity and brain function. These glial cells also play an important role in the development and progression of various neuropathology. Their activation and malfunction occur in unique spatiotemporal patterns, and during these activations, close molecular dialogue is always present between these cells.^85^, ^86^, ^87^, ^88^ Molecular dialogue between microglia and astrocytes in brain development, function, and homeostasis.

The metabolism of microglia and astrocytes is studied relative independently, and there are few studies on the energy interaction between them. It has been found that the growth medium of dense astrocytes culture for three days (ACM) containing CSF‐1/IL‐34, TGF‐β2, and cholesterol can promote the survival of microglia, and microglia recognize and bind to liposomes through TREM2 and transfer cholesterol from astrocytes to microglia is essential for normal physiological function of microglia.^89^ It has also been shown that astrocytes can regulate NO production by microglia by secreting serine and glycine.^90^

**FIGURE 1:**
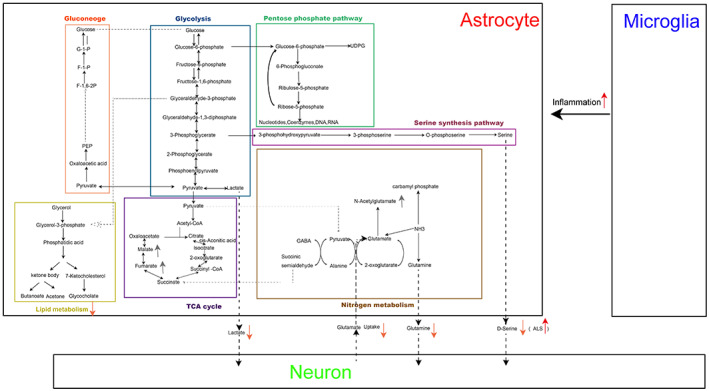
Metabolic changes of astrocytes in neurodegenerative diseases. In neurodegenerative diseases, the exchange of metabolites among astrocytes, neurons, and microglia has changed greatly, which aggravates the occurrence and development of neurodegenerative diseases. Astrocytes lose their homeostasis function. On the one hand, they are reactive to inflammatory factors of microglia. On the other hand, they fail to support the health of neurons. In addition, they also secrete many toxic factors that affect neurons.

In neurodegenerative diseases, the exchange of metabolites among astrocytes, neurons, and microglia has changed greatly, which aggravates the occurrence and development of neurodegenerative diseases. On the one hand, astrocytes are reactive to inflammatory factors of microglia. Upon activation by Aβ, astrocytes participate in the secretion of inflammatory cytokines, thereby contributing to the neurodegenerative changes observed. On the other hand, the lactic acid, glutamine, and d‐serine provided by astrocytes to neurons are reduced, and the glutamate uptake is insufficient, resulting in the inability to support the health of neurons. However, d‐serine supply was enhanced in ALS. d‐serine induces glutamate excitotoxicity in motor neurons and increases glutamate excitotoxicity in both familial and sporadic ALS patients. In addition, they also secrete many toxic factors that affect neurons (Figure 1).

## 5 DYSMETABOLISM OF ASTROCYTES IN NEURODEGENERATIVE DISEASES

### 5.1 Dysmetabolism of astrocytes and aging

Aging means that many physiological changes of body function, including the decline of cell and body function over time, gradually reducing the biological function of cells, the reduction of basic substances of tissues and cells, and the decline of body organ structure and function.^91^ Aging and metabolism are intricately linked. There is strong evidence that aging and aging‐related secretory phenotypes are sensitive to cell metabolic states, which in turn can drive phenotypes related to metabolic dysfunction.^92^ That is, senescent cells are the driving factor of metabolic disorder, which will destroy the metabolic balance of multiple tissues. In fact, metabolic disorders in turn promote aging, thus forming a vicious circle.

Under normal circumstances, the interaction between cell homeostasis and system metabolism is dynamic. With the increase of age, aerobic glycolysis and glucose consumption in the brain are seriously reduced, especially in the temporal, parietal and frontal lobes, and motor cortex. The decrease of brain energy will lead to aging, which is related to the occurrence and development of neurodegeneration. Brain hypometabolism is the first symptom of aging‐related homeostasis disorder.

Astrocyte‐mediated glycolysis is closely related to aging and aging‐related neurodegenerative diseases. Aging is characterized by reduced energy consumption in specific areas of the brain. And astrocytes are the key to regulate neuronal metabolism and neurovascular coupling, so as to consume brain energy for neuronal activity.^93^ Especially, in response to neuronal activity, astrocytes absorb glutamate at synapses, triggering astrocytic aerobic glycolysis, and leading to glucose uptake and lactate release.^21^ Aging survey report shows that, reduced aerobic glycolysis in astrocytes and decreased mitochondrial oxidative phosphorylation in neurons are the main characteristics of aging.^94^, ^95^ On the one hand, neurons mainly rely on mitochondrial oxidative phosphorylation, and their ability to regulate glycolysis and inhibit the accumulation of oxidative stress is limited. Therefore, they are more prone to mitochondrial dysfunction related to aging. On the other hand, astrocyte dysfunction has harmful consequences for neurons, making neurons prone to degeneration, resulting in normal aging to neurodegeneration.^96^ Aging limits the availability of metabolic substrates and the expression of metabolic mechanisms, which is closely related to abnormal energy metabolism, dysfunctional glutamate circulation of astrocytes, and neurovascular coupling changes.^97^ In addition, the decreasing number of astrocytes and the weakened support of neurons through the lactate shuttle are also an important cause of the aging process.

In summary, further dysfunction of astrocytes leads to the progression of aging. These evidences suggest that astrocytes are deeply involved in the vulnerability of the nervous system to the pathological state of aging.

### 5.2 Dysmetabolism of astrocytes in Alzheimer's disease

Alzheimer's disease (AD) is a complex neurodegenerative disorder characterized. It shows a special cognitive and functional decline associated with age together with a neuropathology, including memory loss and language impairment. The pathogenesis of AD is complex and unclear.^98^

**FIGURE 2:**
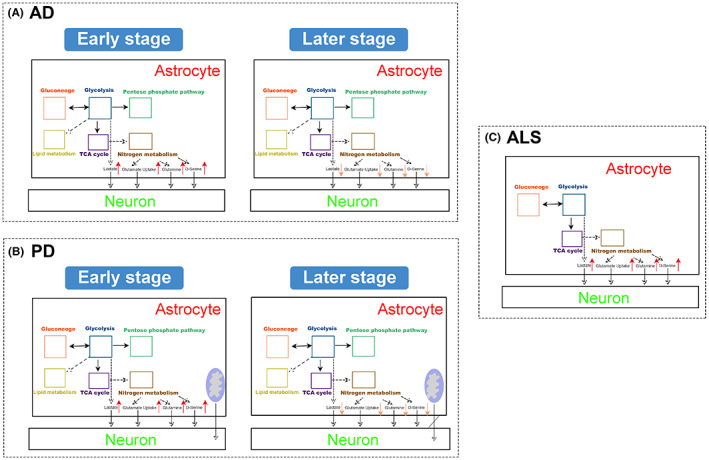
Metabolic changes of astrocytes in AD, PD, and ALS. In the early stage of AD and PD, astrocytes reprogram the metabolic mode to actively response the adverse effects of the disease on neurons, trying to make up for the adverse effects of the pathological process. In the late stage of AD and PD, astrocytes are also damaged by pathological features and cannot compensate for metabolic function.

The effect of astrocytes on Aβ in AD is still a controversy with no consistent answer. Numerous studies have indicated that astrocytes participate in the clearance of Aβ both in vivo and in vitro, suggesting an important role for these astrocytes in the attenuation of the neurodegenerative processes in AD (Figure 2a). For example, astrocytes plated on Aβ‐laden brain sections from an AD mouse model associate with the Aβ deposits and reduce overall Aβ levels in these sections.^99^ Therefore, some articles suggest that the deficits in astroglial clearance of Aβ leads to AD. However, astrocytes can also produce Aβ in some inflammatory states. For instance, TGF‐β1 drives the production of Aβ40/42 by astrocytes leading to Aβ production in TGF‐β1 transgenic mice.^100^ In addition, astrocytes can also rapidly engulf large amounts of Aβ, but then store, rather than completely degrade. The N‐terminal degraded Aβ was produced and partially release to the outside of the cell, causing cortical neuron apoptosis.^101^ The function of dimorphism is closely related to different metabolic patterns.

Jens V. Andersen et al.^102^ found that before Aβ formation, APPswe/PSEN1dE9 mice had altered glucose metabolism, and hampered glutamine processing and mitochondrial. Jens V. Andersen et al.^103^ found that before Aβ formation in 5XFAD mice, reduced astrocyte TCA cycle activity and decreased glutamine synthesis led to hampered neuronal GABA synthesis in the 5xFAD hippocampus. Claudia Salcedo et al.^104^ found that human‐induced pluripotent stem cells (hiPSCs) with APP and PSEN‐1 mutations show reduced leucine‐derived synthesis of glutamate, glutamine, and aspartic acid. The impaired metabolism of branched amino acids may lead to the imbalance of neurotransmitters and energy metabolism in AD. Jens V. Andersen et al.^105^ also found that *de novo* glutamate and glutamine synthesis were reduced in astrocytes in 8 M 5XFAD. Staurenghi Erica et al.^106^ indicated that in the AD brain, astrocyte cholesterol metabolism is abnormal and increased oxysterols promote increased the release of Lcn2, cytokines, and chemokines, ultimately affecting neuronal toxicity. Thus, these all imply that astrocytes are an important component of pathogenesis of AD, and also become an important therapeutic targets of AD. Libin Liu et al.^107^ found that GLP‐1 increased astrocyte support for neurons by activating the PI3K/Akt signaling pathway and alleviating the reduced level of Aβ‐induced astrocyte glycolysis and the production of ROS. However, the development of glia‐aiming drugs remains in a nascent state, and the pathogenesis needs further research.

### 5.3 Dysmetabolism of astrocytes in Parkinson's disease

Parkinson's disease (PD) is the second most common neurodegenerative disorder. Its main pathological features are the loss of dopaminergic (DA) neurons in the dense substantia nigra and the aggregation of intracellular a‐synuclein.^108^ The exact cause for PD is not yet clear, but the underlying molecular mechanisms have been identified in PD pathology. These include a‐synuclein protease stabilization, mitochondrial function, oxidative stress, calcium homeostasis, axonal transport, and neuroinflammation.^109^ There is no cure for PD and currently the treatments are targeted to alleviate motor symptoms by dopamine replacement therapy and surgery.

While PD has been considered as a disease of DA neurons, it is becoming more and more evident that other nonneuronal cell types, including astrocytes, play an important role in the pathogenesis of PD (Figure 2b). Several genes known to have a causative role in the development of PD, such as PARK2, PARK7, PINK1, LRRK2, SNCA, ATP13A2, PLA2G6, and GBA are highly expressed in astrocytes and affect astrocyte metabolism:

PINK1, Parkin, GBA, and DJ‐1 have all been shown to have a role in the maintaining of healthy mitochondria.^110^, ^111^, ^112^, ^113^ IPLA2 regulate the release of fatty acid arachidonic acid (AA) from phospholipids, and ATP‐mediated calcium response.^114^, ^115^ A‐synuclein (a‐SYN) regulates the incorporation and distribution of AA and palmitic acid,^116^ and glutamate transporter GLST1 and GLT1 expression and BBB function, and water transport of aquaporin 4(AQP4) localization.^117^ DJ‐1 regulate the degradation of lipid rafts protein flotillin‐1 and caveolin‐1 and affect the assembly of lipid rafts (lipid rafts), thereby maintaining the glutamate transporters GLAST on the cell membrane.^118^ Parkin regulate the secretion of glutathione in astrocytes and affect neurotrophic ability.^119^, ^120^ DJ‐1 terminate TLR4 signaling pathway through receptor endocytosis and inhibit IFN‐g inflammatory response.^121^ GBA regulates the expression of cathepsin lysosomal proteases,^122^ LRRK2 and ATP13A2 regulate the PH of lysosomal,^123^, ^124^ LRPK2 affects LC3 lipidation,^124^ ATP13A2 maintains lysosome stability,^125^, ^126^ all above jointly regulate protein degradation. Parkin and PINK1 regulate astrocyte proliferation, which, in the case of PINK, function by regulating EGFR protein levels through AKT/p38‐dependent pathways.^119^, ^120^, ^123^

Previous research^127^ showed that vitamin d‐activating enzyme CYP27B1 positive astrocytes could display neuroprotective features as they sequester α‐Synuclein oligomers and are associated with Lewy body negative neurons.

Although there are few studies on the role of astrocytes in the pathogenesis of PD compared with the study of neuronal function, indications of astrocytes' involvement in PD have begun to emerge. Samanta Mazzetti et al. revealed that CYP27B1 is increased in astrocyte subpopulations with neuroprotective characteristics in PD patients, and that CYP27B1 positive astrocytes are involved in autophagy‐mediated ‐α‐synuclein uptake. Vitamin D can treat PD by preventing astrocyte changes.^127^ Yuqi Zhang et al.^128^ suggest that astrocytes regulate neuronal synaptic plasticity by releasing adenosine triphosphate, glutamate, and d‐serine, which may provide a new direction for the treatment of PD.

### 5.4 Dysmetabolism of astrocytes in ALS

Astrocytes play a crucial role in the pathogenesis of both sporadic and hereditary familial ALS which is associated with the mutation of the human superoxide dismutase 1 (hSOD1) gene. In hSOD1/G93A ALS model mice, astrocytes undergo morphological and pathological remodeling, loss of function, and cell death. The changes of astrocytes further lead to the aggravation of neuronal abnormalities and the appearance of clinical symptoms.^129^ Selective silencing of hSOD1 gene in astrocytes delays the progression of ALS.^129^

Oliver J Ziff et al.^130^ showed that astrocytes in ALS are characterized by upregulation of genes related to extracellular matrix, endoplasmic reticulum stress, and immune response, and downregulation of genes related to synaptic integrity, glutamate uptake, and neural support (Figure 2c). These results suggest that astrocytes in ALS enhance inflammatory processes and inhibit neuronal support mechanisms. The key pathogenic factor associated with neurotoxicity is insufficient glutamate uptake by astrocytes. A fundamental function of astrocytes is to control and reduce the concentration of extracellular glutamate. Excessive glutamate in synaptic space, which is toxic to neurons, can lead to excessive discharge of neurons and increase calcium influx. In ALS mice, glutamate is also released by microglia.^131^, ^132^ Therefore, the neurotoxicity of glutamate in ALS is closely related to neuroinflammation. Another neurotoxicity molecule closely related to astrocytes in ALS is d‐serine, which is an endogenous comonomer of NMDA receptor. d‐serine induces glutamate excitotoxicity in motor neurons and increases glutamate excitotoxicity in both familial and sporadic ALS patients.^133^ In addition, mutations in the gene encoding d‐amino acid oxidase (which controls the level of d‐serine) are associated with many cases of ALS families.^134^

Mitochondrial dysfunction is a key mechanism of motor neuron degeneration in ALS. In addition to motoneurons, mitochondrial abnormalities in astrocytes lead to elevated reactive oxygen species (ROS) levels, which is associated with neurodegeneration in SOD1‐ALS mice.^135^ Similarly, increased levels of NADPH oxidase (NOX2), inducible nitric oxide synthase (iNOS), and ROS were observed in human astrocytes expressing mutant SOD1, which induced non‐cellular autonomic toxicity in human ES cell‐derived motor neurons.^136^ Consistent with these observations, in the sod1‐als model, an experimental treatment for overexpression of Nrf2 (nuclear factor erythroid‐2 related factor 2), a transcription factor, upregulates genes encoding antioxidants, especially in astrocytes.

In addition, Stella Robertoh et al. found changes in lipid metabolism in primary astrocytes in SOD1 (G93A), characterized by an increase in FAS and a reduction in the two intermediates of glycerol metabolism (i.e., galactosyl glycerol and phospholipic acid). These results indicate the changes of lipid metabolism/signal transduction in astrocytes of ALS.^137^ Madji Hounoum Blandine et al. showed that compared with those cultured alone, astrocytes co‐cultured with motor neurons upregulated the glutamate and induced the expression of glutamate transporter (GLT/EAAT2), increased the capacity of glutamate/aspartic acid transporter (GLAST/EAAT1) and most metabolites involved in purine and pyrimidine pathways. However, compared with wild‐type motor neuron co‐cultured astrocytes，the accumulation of glutamate in SOD1G93A astrocytes indicated that dysregulation of the glutamate‐glutamine cycle, and SOD1G93A astrocytes treated with glutamate showed decreased levels of lactic acid, creatine, creatinine, deoxycarnitine, and l‐acetylcarnitine.^138^ Therefore, astrocytes in ALS have metabolic dysfunction, and these metabolic pathways can be used to open up new therapeutic targets for ALS.

## AUTHOR CONTRIBUTIONS

L.LG., and J.Z. conceptualized the study and wrote the manuscript. C.ZL., Y.ZQ., Y.SC. and X.MQ. discussed and edited the manuscript. L.LG. supervised the project. All authors reviewed and gave final approval to the manuscript.

## FUNDING INFORMATION

National Nature Science Foundation of China Grant 81,801,337 (L.L.). National Nature Science Foundation of China Grant 82,071,520 (L.L.). Fujian Province Nature Science Foundation (Grant: 2019 J05006 to L.L.). Xiamen Youth Innovation Fund Grant 3502Z20206031 (L.L.).

## CONFLICT OF INTEREST

The authors declare no competing interests.

## Data Availability

The data that support the findings of this study are openly available.
